# *TBX21* and *HLX1* Polymorphisms Influence Cytokine Secretion at Birth

**DOI:** 10.1371/journal.pone.0031069

**Published:** 2012-01-30

**Authors:** Vera Isabel Casaca, Sabina Illi, Kathrin Suttner, Isolde Schleich, Nikolaus Ballenberger, Elizabeth Klucker, Elif Turan, Erika von Mutius, Michael Kabesch, Bianca Schaub

**Affiliations:** 1 Department of Pulmonary and Allergy, University Childreńs Hospital Munich, Munich, Germany; 2 Department of Paediatric Pneumology, Allergy and Neonatology, Hannover Medical School, Hannover, Germany; University of Tübingen, Germany

## Abstract

**Background:**

TBX21 (T cell specific T-box transcription factor) and HLX1 (H.20-like homeobox 1) are crucial transcription factors of T_H_1-cells, inducing their differentiation and suppressing T_H_2 commitment, particularly important for early life immune development. This study investigated the influence of *TBX21* and *HLX1* single nucleotide polymorphisms (SNPs), which have previously been shown to be associated with asthma, on T_H_1/T_H_2 lineage cytokines at birth.

**Methods and Findings:**

Cord blood mononuclear cells (CBMCs) of 200 neonates were genotyped for two *TBX21* and three *HLX1* SNPs. CBMCs were stimulated with innate (Lipid A, LpA; Peptidoglycan, Ppg), adaptive stimuli (house dust mite *Dermatophagoides pteronyssinus* 1, Derp1) or mitogen (phytohemagglutinin, PHA). Cytokines, T-cells and mRNA expression of T_H_1/T_H_2-related genes were assessed. Atopic diseases during the first 3 years of life were assessed by questionnaire answered by the parents.

Carriers of *TBX21* promoter SNP rs17250932 and *HLX1* promoter SNP rs2738751 showed reduced or trendwise reduced (p≤0.07) IL-5, IL-13 and TNF-α secretion after LpA-stimulation. Carriers of *HLX1* SNP rs2738751 had lower IL-13 levels following Ppg-stimulation (p = 0.08). Carriers of *HLX1* exon 1 SNP rs12141189 showed increased IL-5 (LpA, p = 0.007; Ppg, p = 0.10), trendwise increased IL-13 (LpA), higher GM-CSF (LpA/Ppg, p≤0.05) and trendwise decreased IFN-γ secretion (Derp1+LpA-stimulation, p = 0.1). Homozygous carriers of *HLX1* promoter SNP rs3806325 showed increased IL-13 and IL-6 (unstimulated, p≤0.03). In carriers of *TBX21* intron 3 SNP rs11079788 no differences in cytokine secretion were observed. mRNA expression of T_H_1/T_H_2-related genes partly correlated with cytokines at protein level. *TBX21* SNP rs11079788 carriers developed less symptoms of atopic dermatitis at 3 years of age (p = 0.03).

**Conclusions:**

Polymorphisms in *TBX21* and *HLX1* influenced primarily IL-5 and IL-13 secretion after LpA-stimulation in cord blood suggesting that genetic variations in the transcription factors essential for the T_H_1-pathway may contribute to modified T_H_2-immune responses already early in life. Further follow-up of the cohort is required to study the polymorphisms' relevance for immune-mediated diseases such as childhood asthma.

## Introduction

Asthma and inflammatory diseases are induced by a complex interplay of genetic and environmental factors influencing early immune responses [1], [2], [3]. Modulation of the immune system in early life via innate stimuli may play an essential role in preventing allergic responses [4], as shown in farm children, which are exposed to microbial innate stimuli and have a lower prevalence of allergic diseases [5].

T_H_1/T_H_2 imbalance can lead to different inflammatory conditions and has been described in several immune-mediated diseases including asthma [6]. T_H_1 and T_H_2 cell lineages are controlled by cell-specific transcription factors (TFs) [7]. TBX21, is a specific TF of T_H_1-cells, which promotes their differentiation and proliferation and suppresses T_H_2-cell development. It activates the expression of the T_H_1 hallmark cytokine INF-γ and represses IL-4 production in developing T_H_2-cells [8]. *TBX21* polymorphisms were associated with airway hyperresponsiveness in asthma [9] and increased risk for childhood asthma [10]. TBX21 interacts closely with HLX1, another T_H_1 transcription factor. The interaction between TBX21 and HLX1 is required to induce maximal IFN-γ secretion [11]. Both genes have the capacity to revert T_H_2 cell commitment of T cells already expressing T_H_2 cytokines [11]. Further downstream activation of T_H_1 and T_H_2 cytokines is regulated tightly by these transcription factors [8], [12], [13]. Like *TBX21*, polymorphisms in *HLX1* have also been significantly associated with the development of childhood asthma [14]. Functional studies on some of these genetic variants already demonstrated their putative functional relevance [10], [14]. In general, lineage-specific TFs control other TFs and genes that encode for cytokines [7].

Cytokine regulation has shown to be crucial for later development of immune-mediated diseases such as asthma. Thus, it is of particular interest in this context to study early life immune modulation. Cytokines as important mediators in the development of allergies are already modulated during pregnancy, and a T_H_2-dominated cytokine pattern can be detected in cord blood [15], [16], [17], [18].

We hypothesized that polymorphisms in these T_H_1-specific TFs influence T cell immune responses already at birth. We aimed to study two *TBX21* (rs17250932, rs11079788) and three *HLX1* (rs2738751, rs3806325, rs12141189) polymorphisms, which have previously been associated with risk or protection from childhood asthma. We investigated whether these SNPs have an impact on cytokine secretion patterns and mRNA expression of T_H_1/T_H_2-related genes. We further assessed the impact of the atopic status of the mother as strong predictor for the development of childhood allergic diseases [15]. Additionally, we investigated the development of atopic diseases of the children during the first 3 years of life.

## Methods

### Ethics Statement

Written informed consent was obtained from the mothers for participation. Approval was obtained from the local human research committee of the Bavarian Ethical Board, LMU Munich, Germany.

### Population characteristics

Fetal cord blood was obtained from neonates (n = 200) born in the Munich metropolitan area, Germany. Informed consent was given by the mothers for participation in the study, including cord blood collection. The enrolment period was from July 2005 until September 2007. Inclusion criteria comprised healthy neonates and mothers with uncomplicated pregnancies. Exclusion criteria included preterm deliveries and perinatal infections. Maternal atopy was defined as doctor's diagnosis of asthma and/or eczema and/or hay fever. In addition, total and specific IgE (Radio-Allergo-Sorbent Test) was measured. A positive specific IgE was defined as 1 or more positive reactions (≥0.35 IU/ml) to a panel of 20 common allergens.

### Follow-up at age 3 years

A follow-up at the age of 3 years was performed. The data were assessed by detailed questionnaires answered by the parents. Wheeze was defined by wheezy symptoms in the first 3 years. Atopic dermatitis (AD) was defined by symptoms of AD in the first 3 years, and skin manifestations of food allergy were noted. A positive allergy test was defined by parental report of a positive skin Prick test or blood test with at least one positive test to one of the 20 common inhalant or food allergens.

### Polymorphisms selection and genotyping

Based on previous studies, 5 polymorphisms in *TBX21* and *HLX1* were selected for this analysis due to their association with asthma and their putative functional implications [10], [14]. Genotyping was performed by Matrix-assisted laser desorption/ionization time-of-flight mass spectrometry (Sequenom Inc., San Diego, CA, USA). Polymerase chain reaction assays and associated extension reactions were designed with the SpectroDESIGNER software (Sequenom Inc.). All amplification and extension reaction conditions have been previously described [19]. Deviations from Hardy–Weinberg Equilibrium were assessed for quality control of genotyping procedures (Table 1). Of note, wild type (WT) refers to WT alleles in absence of the particular polymorphic allele under study.

**Table 1:** Characteristics of the studied *TBX21* and *HLX1* polymorphisms.

Gene	rs No.	Position in relation to 1.ATG	Position in the gene structure	MAF (ISAAC+)	MAF (our cohort)	P value HWE
*TBX21*	rs17250932	T−1514Cψ	Promoter	0.16	0.22	0.5956
*TBX21*	rs11079788	C+9902Tψ	Intron 3	0.22	0.30	0.8273
*HLX1*	rs2738751	C−1486G*	Promoter	0.14	0.15	0.8804
*HLX1*	rs3806325	C−1407T*	Promoter	0.19	0.18	0.4744
*HLX1*	rs12141189	T+346C* Δ	Exon 1	0.25	0.25	0.4025

*Based on the National Center for Biotechnology Information GenBank sequence (accession no. AF217621).

ψ Based on the *TBX21* sequence obtained from the SNPper database (http://snpper.chip.org).

+ German children (total n = 3099) from the cross-sectional International Study of Asthma and Allergy in Childhood phase II.

Δ SNP leads to an amino acid change. HWE = Hardy–Weinberg Equilibrium. MAF, Minor Allelle Frequency. HWE, Hardy-Weinberg Equilibrium.

### Real-time quantitative RT-PCR

Total RNA was isolated from CBMCs with TRI reagent, cDNA synthesis was performed using reverse transcriptase (Invitrogen, Karlsruhe, Germany). Vector NTI advance10 (Invitrogen, Karlsruhe, Germany) was used for specific primer pairs design (18S, GATA3, STAT6, STAT6e, TBX21, HLX1, IRF1). Gene-specific PCR-products were measured continuously by means of iCycler Real-Time PCR-Detection-System (Bio-Rad) for 40 cycles. Direct detection of the PCR product was monitored by measuring the increase in fluorescence caused by the binding of SYBR Green to dsDNA. The threshold cycle (ct) of each target product was determined and set in relation to the amplification plot of 18S (Δct). The level of mRNA of each gene is described as gene expression.

### Cytokine secretion

CBMCs, isolated freshly within 24 hours by Ficoll-Hypaque density gradient, were stimulated with Lipid A (LpA, 0.1 µg/ml), Peptidoglycan (Ppg, 10 µg/ml), allergen house dust mite (Derp1, 30 µg/ml), a combination of Derp1 and LpA (D+L) or phytohemagglutinin (PHA, 5 µg/ml) for 3 days and compared with unstimulated cells as previously described [15]. Cytokine concentrations were measured in supernatants by using the Human Cytokine-Multiplex-Assay-Kit according to the manufacturer's instructions (Bio-Rad, Munich, Germany) by LUMINEX technology. The lower limits of detection (pg/ml) were 1.8 (IL-5), 0.5 (IL-6), 3.0 (TNF-α), 0.21 (IL-13), 1.3 (IFN-γ), 1.0 (GM-CSF). Non-detectable cytokine concentrations were assigned to a value of 0.01 for inclusion into the analysis.

### Flow cytometry

Cells were analyzed by using 3-color flow cytometry (FACScan; BD Biosciences, Heidelberg, Germany). For surface staining, 2 µl of anti-human CD4–fluorescein isothiocyanate (FITC) and 1 µl of CD25–RPE-Cy5 (Dako Cytomation, Glostrup, Denmark), were used. For isotype control 1 µl of IgG1-FITC (Dako Cytomation, Glostrup, Denmark), and 0.5 µl of IgG2a RPE-Cy5 (BD Biosciences) were added [4]. Data were analyzed with CellQuest software (BD Biosciences), and postacquisition analysis was performed with WinMDI 2.8 software (The Scripps Research Institute, La Jolla, CA).

### Statistical analysis

Non-parametric tests (Wilcoxon/Kruskal-Wallis) were used to compare the median of cytokine concentrations, as these were generally not normally distributed and could not be transformed to normality. Data were reported as median, first and third quartile. Chi^2^ tests were used to evaluate categorical predictor variables. Data for genetic analyses were analyzed with Kruskal-Wallis-test (three groups). Based on previous reports which described recessive effects for T_H_1-related SNPs, we have further analysed the SNP effects using a recessive model. Due to the fact that gene expression variables contained non-detectable measurements (censored data), summary statistics such as the median and quartiles was conducted by the Kaplan-Meier method [20]. Testing on group differences was performed by the generalized Wilcoxon test [21]. Data were not adjusted for multiple testing as this is an explorative study. In order to assess the role of maternal atopy we performed stratified analysis for the CBMCs of the neonates with or without maternal atopy. Statistical significance was defined by p≤0.05; data analysis was performed with SAS 9.2 (The SAS Institute, Cary, NC, USA).

## Results

We assessed the effect of 5 genetic variants located in T_H_1 lineage transcription factors on cord blood immune responses of 200 neonates, namely *TBX21* (rs17250932 and rs11079788) and *HLX1* (rs2738751, rs3806325 and rs12141189) (Table 1) which have previously been related to the development or protection from asthma [10], [14]. The genotyping success rate was at least 97%. None of the polymorphisms deviated significantly from Hardy-Weinberg Equilibrium.

### T_H_2-related cytokine secretion upon innate stimulation was modulated in carriers of *TBX21* and *HLX1* polymorphisms

**Figure 1:**
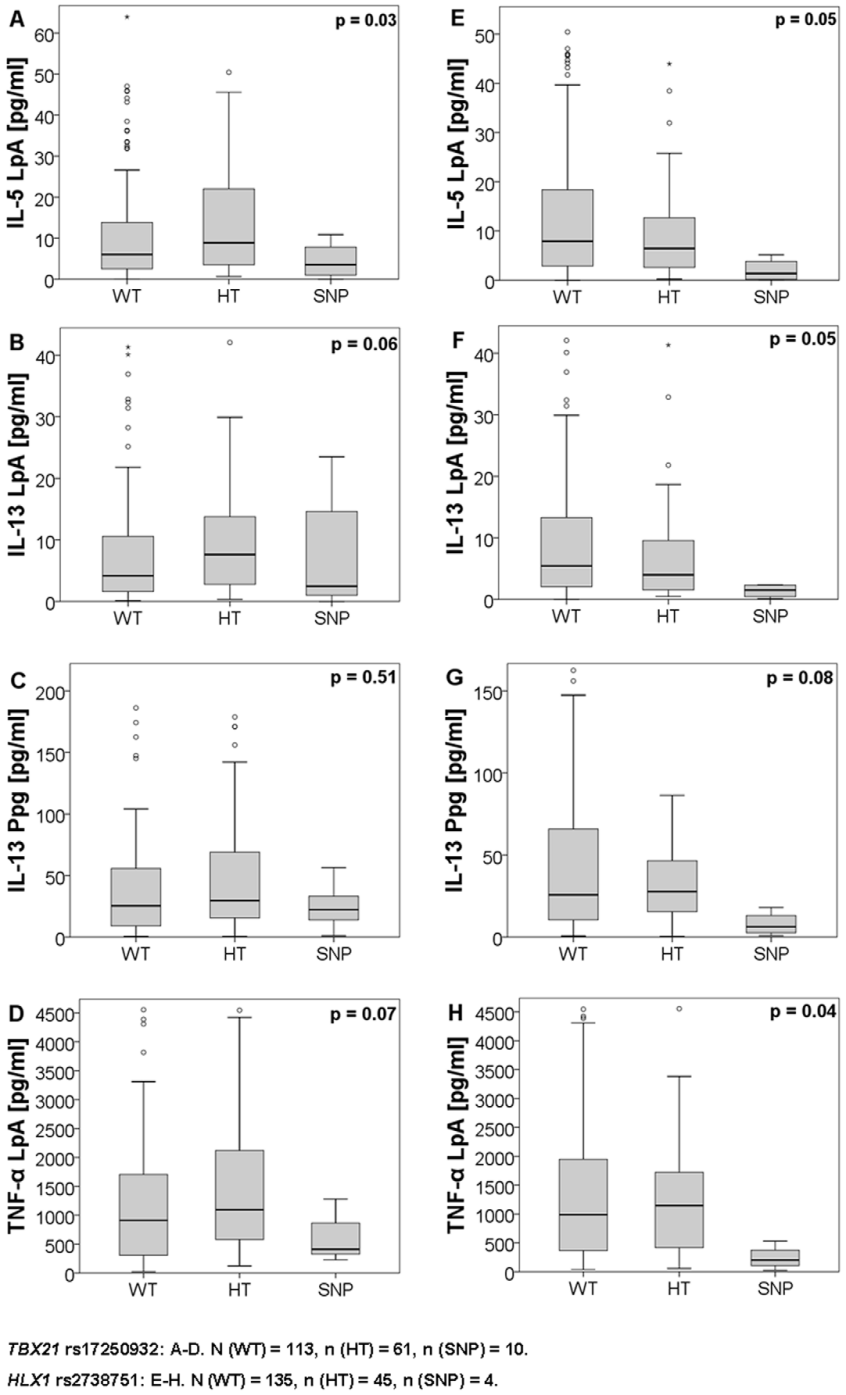
Cytokine secretion of wildtype, heterozygous and homozygous SNP carriers of *TBX21* rs17250932 and *HLX1* rs2738751. Data were shown in boxplots (first, third quartile, median), the whiskers indicate the maximum and minimum values, dots indicate outliers, analyzed by Kruskal-Wallis-test. Values were shown in pg/ml. n (WT) = 113, n (HT) = 61, n (SNP) = 10, and *HLX1* rs2738751 n (WT) = 135, n (HT) = 45, n (SNP) = 4.

Assessment of cytokine responses in CBMCs showed that carriers of *TBX21* SNP rs17250932 (promoter) and *HLX1* SNP rs2738751 (promoter) had decreased or trendwise decreased IL-5 and IL-13 after LpA and for the latter also decreased IL-13 after Ppg-stimulation compared to wild-type (WT) and heterozygous carriers (HT). TNF-α secretion was trendwise lower or significant lower in carriers of *TBX21* SNP rs17250932 and *HLX1* SNP rs2738751, respectively (Fig. 1).

**Figure 2:**
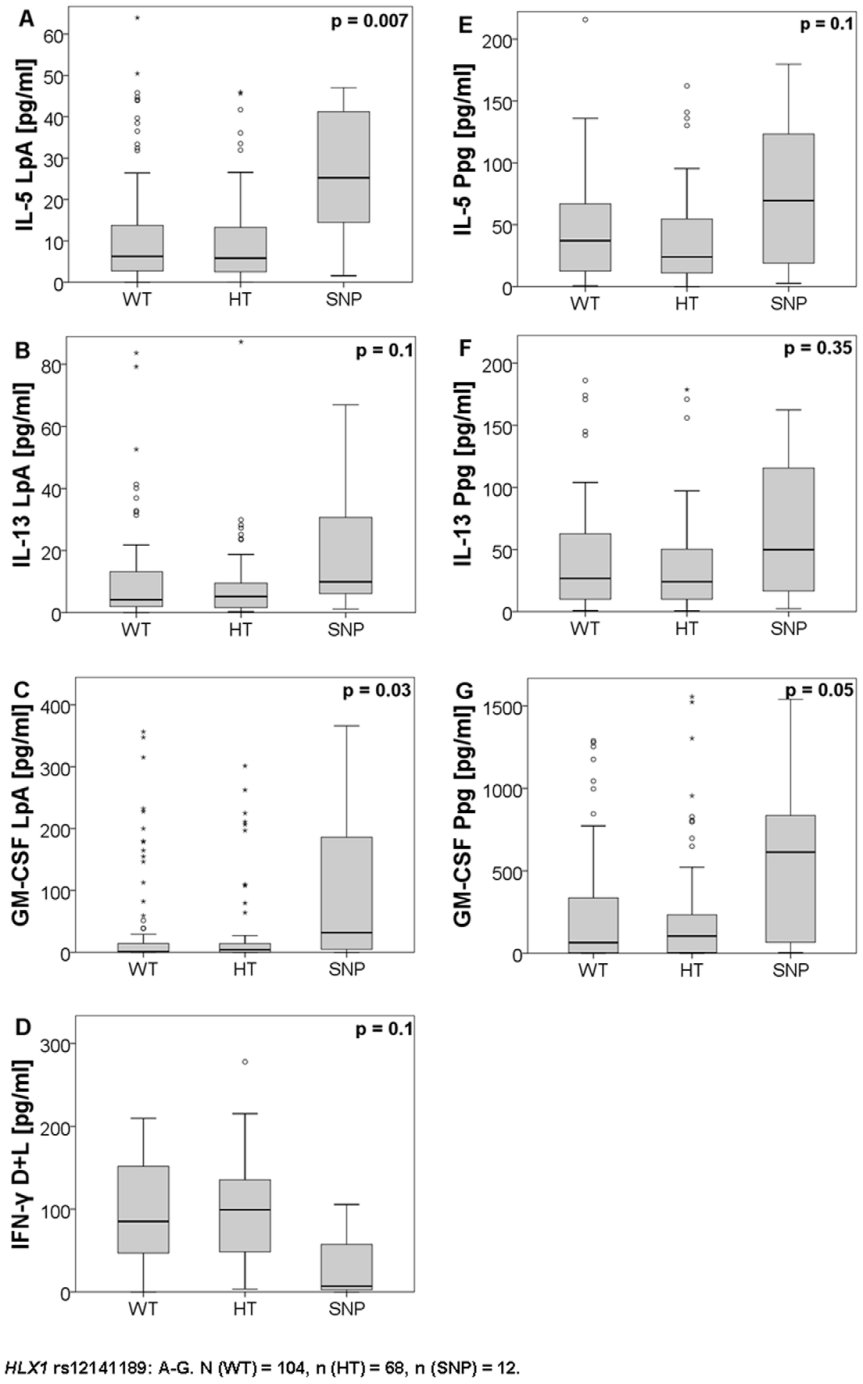
Cytokine secretion of wildtype, heterozygous and homozygous SNP carriers of *HLX1* rs12141189. Data were shown in boxplots (first, third quartile, median), the whiskers indicate the maximum and minimum values, dots indicate outliers, analyzed by Kruskal-Wallis-test. Values were shown in pg/ml. n (WT) = 104, n (HT) = 68, n (SNP) = 12.

On the other hand, increased IL-5, IL-13 and GM-CSF secretion was observed in the carriers of *HLX1* SNP rs12141189 (exon 1), after LpA and Ppg-stimulation, while the T_H_1-cytokine IFN-γ was trendwise downregulated after D+L-stimulation (Fig. 2). Additionally we analyzed the polymorphisms using a recessive model, as previous reports described recessive effects for T_H_1-related SNPs. After applying the recessive model, the majority of the findings achieved greater statistical significance (Table 2).

**Table 2:** Effects of *TBX21* and *HLX1* polymorphisms on cytokine secretion, T cells and mRNA regulation.

Gene/rs number	Cytokine secretion/T cell regulation	P Overall	P recessive model	mRNA regulation	P Overall
*TBX21* rs17250932	IL-5 (LpA)↓	**0.03**	**0.03**	No changes with LpA	-
	IL-13 (LpA)↓	0.06	0.28	*STAT6e* (U)↓	**0.01**
	TNF-α (LpA)↓	0.07	0.16		
*TBX21* rs11079788	CD4^+^CD25^+^ (U)↑	**0.04**	0.23	*GATA3* (U)↑	0.08
				*HLX1* (U)↑	**0.02**
				*IRF1* (U)↑	**0.01**
*HLX1* rs2738751	IL-5 (LpA)↓	**0.05**	**0.02**	No changes with LpA or Ppg	-
	IL-13 (LpA)↓	**0.05**	**0.02**		
	IL-13 (Ppg)↓	0.08	**0.03**		
	TNF-α (LpA)↓	**0.04**	**0.01**		
*HLX1* rs3806325	IL-13 (U)↑	**0.005**	**0.003**	*GATA3* (LpA)↑	**0.003**
	IL-6 (U)↑	**0.03**	**0.05**	*STAT6e* (LpA)↑	**0.007**
*HLX1* rs12141189	IL-5 LpA↑	**0.007**	**0.002**	*TBX21* (D+L)↓	**0.02**
	IL-13 LpA↑	0.1	**0.04**		
	GM-CSF LpA↑	**0.03**	**0.009**		
	IL-5 (Ppg)↑	0.1	0.09		
	GM-CSF (Ppg)↑	**0.05**	**0.02**		
	IFN- γ (D+L)↓	0.1	**0.03**		

LpA = Lipid A; Ppg = Peptidoglycan and U = unstimulated, D+L = *Dermatophagoides pteronyssinus* 1 and Lipid A;

↑ : expression upregulated;

↓ : expression downregulated. *P* values analyzed by Kruskal-Wallis-test (overall, 3 categories) or Wilcoxon-test (recessive model, 2 categories). Genotype comparison includes the comparison of the respective genotype groups used for statistical analysis of mRNA expression; statistics performed by generalized Wilcoxon test. Significance (p≤0.05) is marked in bold.

Carriers of *HLX1* SNP rs3806325 (promoter), showed increased IL-13 and IL-6 levels in unstimulated cells (Table 2), and no significant changes were observed for other cytokines or following stimulation.

Carriers of the *TBX21* SNP rs11079788 (intron 3) showed significantly higher CD4^+^CD25^+^ cells (2.79%) in comparison to the HT (2.38%) and WT (1.78%) in unstimulated cells. However no significant changes in cytokine secretion were detected as for the other four studied T_H_1-related polymorphisms. No statistically differences were observed following PHA-stimulation for any of the polymorphisms. Previously we have shown that two *TLR*2 polymorphisms were important for the modulation of T regulatory cells at birth [22]. Assessing whether these *TLR*2 polymorphisms have an additional effect in this study, we detected only 1–3 children with homozygous polymorphisms for both *HLX1* and *TLR2* (Table S1). Thus, we can not disentangle potential effects of *TLR2* polymorphisms in our study.

### Carriers of *TBX21* and *HLX1* polymorphisms showed modulated mRNA expression of T_H_1/T_H_2 related genes

We assessed the effects of the SNPs on mRNA expression of T_H_1/T_H_2 related genes, namely *TBX21*, *HLX1*, *IRF1*, *GATA3*, *STAT6 and STAT6e*. We included gene expression data related to the main findings at cytokine protein level. Carriers of *TBX21* SNP rs17250932 showed lower *STAT6e* mRNA expression, in unstimulated cells although the decrease at protein level of T_H_2/proinflammatory cytokines was observed after innate stimulation (Table 2)t. Carriers of *TBX21* rs11079788 had increased T_H_2/T_H_1 related genes *GATA3* and also increased *HLX1* and *IRF1* mRNA expression in unstimulated cells, in parallel with increased activated T cells (Table 2).

*HLX1* rs3806325 SNP carriers showed increased *GATA3* and *STAT6e* mRNA expression following LpA-stimulation, in parallel with higher IL-13 and IL-6 cytokine response, the latter in unstimulated cells. *HLX1* rs12141189 SNP carriers showed lower *TBX21* gene expression (D+L), in parallel with lower IFN-γ protein secretion. No significant changes at mRNA level were detected for *HLX1* rs2738751 SNP carriers (Table 2).

### Maternal atopy showed no additional effect on the SNP-induced immune modulation

Due to the relevance of maternal atopy on cord blood immune responses [15], we further assessed whether the modulated immune responses in SNP carriers were influenced by the atopic status of the mother. However, in stratified analysis all observed effects were of similar magnitude for children with and without maternal atopy indicating no effect of maternal atopy in the present study (data not shown). In addition, the Minor Allelle Frequencies (MAF)of *TBX21* and *HLX1* SNPs differed only sligthly in the CBMC of neonates from either atopic or non-atopic mothers (Table 3).

**Table 3:** Minor Allele Frequency (MAF) in children of atopic and non-atopic mothers.

Gene/rs number	Maternal atopy	MAF
*TBX21* rs17250932	No	0.25
	Yes	0.17
*TBX21* rs11079788	No	0.32
	Yes	0.25
*HLX1* rs2738751	No	0.15
	Yes	0.15
*HLX1* rs3806325	No	0.16
	Yes	0.21
*HLX1* rs12141189	No	0.20
	Yes	0.23

Minor Allele Frequency of *TBX21* and *HLX1* polymorphisms in children of atopic and non atopic mothers.

### Impact of *TBX21* and *HLX1* polymorphisms on health outcomes

The cohort was followed up to the age of 3 years and symptoms of atopic diseases and wheeze were assessed by questionnaires. The distribution of children that developed atopic diseases within the different *TBX21* and *HLX1* genotypes (WT, HT and SNP) is described in the following: homozygous carriers of *TBX21* rs11079788 SNP showed less symptoms of atopic dermatitis (19%) compared to HT (23%) and WT (36%) (p = 0.03). No significant associations were observed for the other *TBX21* and *HLX1* polymorphisms with wheeze, skin manifestation of food allergy or atopic dermatitis at this time point.

## Discussion

In this study, we assessed the effect of single nucleotide polymorphisms (SNPs) of T_H_1 transcription factors, namely TBX21 and HLX1 on T cell lineages and in particular T_H_1/T_H_2 cytokine secretion in cord blood. Carriers of *TBX21* and *HLX1* polymorphisms showed modulated IL-5 and IL-13 (T_H_2) cytokine secretion upon innate stimulation with Lipid A or peptidoglycan in cord blood. T_H_1/T_H_2-related mRNA expression partly correlated with cytokine secretion.

The influence of a child's genotype on the development of immune-mediated diseases such as allergic diseases has been shown in several studies, e.g. for *IRF1* or *TLR* polymorphisms [22], [23], [24]. For *TBX21* polymorphisms, several studies have shown an association with the development of asthma [25], [26], [27] indicating the relevance of T_H_1 TFs in T_H_2-associated allergic diseases. Therefore we investigated the effect of SNPs in the T_H_1 pathway (*TBX21* and *HLX1*) on primarily T_H_1 and T_H_2 cytokine regulation early in life, a critical time window, which is relevant for early immune maturation and subsequent determination of T_H_2-mediated allergic diseases [28], [29], [30]. We detected a decrease of IL-5 and a trend for decreased IL-13 secretion after LpA-stimulation in homozygous carriers of *TBX21* SNP rs17250932. This decrease may potentially be explained by an overexpression of *TBX21*. Indeed, it has previously been shown experimentally that this polymorphism increases promoter activity [10]. Szabo et al. have shown that expression of *TBX21* in T_H_2-cells by retroviral gene transduction leads to lower IL-4 and IL-5 [8]. Furthermore, IL-13 mRNA expression and its promoter activity were suppressed upon *TBX21* expression suggesting a regulation of IL-13 at gene transcription level [12]. In parallel, T-bet null mice showed elevated production of IL-4, IL-5 and IL-13, and diminished production of interferon-γ [31]. In unstimulated conditions the *TBX21* SNP rs17250932 carriers showed reduced *STAT6e* mRNA expression, which represents a *STAT6* isoform (splice variant including both intron 17 and intron 18) [32]. The second *TBX21* SNP (rs11079788) investigated in our study was not associated with any change in cytokine responses. Yet, homozygous carriers had a higher number of activated T cells, combined with an increased expression of *GATA3*, *HLX1*, *IRF1* mRNA expression, representing T_H_1 as well as T_H_2-genes.

Similar to *TBX21* rs17250932, homozygous carriers of *HLX1* SNP rs2738751, showed lower T_H_2 and inflammatory cytokine secretion (IL-5, IL-13 and TNF-α) after LpA-stimulation.

Carriers of *HLX1* polymorphism rs12141189 showed increased T_H_2 cytokines such as IL-5, IL-13 and also GM-CSF following innate stimulation (LpA or Ppg). In parallel to higher T_H_2 cytokines, we detected also lower T_H_1 IFN-γ secretion and lower *TBX21* mRNA expression. This could lead to *HLX1* dowregulation, as HLX1 can be induced by TBX21 [11]. This increase of T_H_2 cytokines at birth in our study may potentially be in contrast with an asthma-protective effect observed in school-age children [14]. Although without stimulation no differences at mRNA-level of T_H_1/T_H_2-related genes were detected, the LpA-induced increase of *GATA3* and *STAT6* mRNA expression in homozygous carriers of the *HLX1* SNP rs3806325 may be supportive of a T_H_2-dominated immune response. However different influences on immune maturation during childhood may modify the T cell lineage fate over time. Thus, a direct comparison between findings in cord blood and school-age children needs to be interpreted with caution.

Homozygous carriers of *HLX1* SNP rs3806325, which was previously associated with an enhanced risk for asthma, especially non-atopic asthma in childhood (14), showed increased levels of IL-13 (T_H_2) and additionally enhanced IL-6 secretion in this cord blood study. Previous functional studies have demonstrated that this polymorphism significantly influences *HLX1* gene expression levels due to an altered transcription factor binding to the *HLX1* promoter, indicating its potential functional relevance [14]. Results comparing *HLX1* SNP carriers *vs* heterozygous and WT carriers became more significant when applying the recessive model, which compares the homozygous SNP carriers *vs* heterozygous carriers and wild type. Overall, immune development is influenced by a variety of factors over time, and cord blood responses may not be translated to childhood atopic phenotypes without taking additional regulatory factors into account. It is likely that certain genotypes may be more relevant than others and that the interplay with specific environmental exposures will impact T_H_ cell lineages and disease development differently.

In our study most of the effects of *TBX21* and *HLX1* polymorphisms of T_H_1 pathway were observed upon LpA-stimulation, partly upon Ppg-stimulation and almost no differences were observed following Derp1-stimulation. Thus, the effects seem to be influenced by innate immune regulation, mainly via TLR4-stimulation. For further functional explanation, more detailed studies are required.

Furthermore, we described in our previous studies that maternal atopy modulated cord blood immune responses [15], [22]. Yet, in stratified analysis in this study, all observed effects were of similar magnitude for children with and without maternal atopy.

In regards to allergic outcomes assessed at age 3 years, one *TBX21* polymorphism was associated with less symptoms of atopic dermatitis. Therefore, further investigations on both *TBX21* and *HLX1* polymorphisms are required in larger cohorts to confirm the effects of the polymorphisms on atopic diseases in childhood. It may be of particular interest to investigate children at risk for atopic diseases, in order to disentangle the effects of the SNPs on early immune development and subsequent disease.

Additional points need to be considered for interpretation of our data. The results were not adjusted for multiple testing as this is an explorative study and further replication by other studies rather than correction for multiple testing is required. Another important point is the assessment of cytokines in bulk culture due to logistic possibilities and low cell numbers *per* child. Thus, we were not able to clearly differentiate the origin of the secreting cell. Additionally, we do not have details about leukoyte subsets available. The low frequency of SNPs resulted in low numbers of homozygous carriers of the SNPs in our cohort, therefore some findings could possibly be masked or power was limited due to the number of affected children.

The strength of this study is a detailed investigation of the early immune system regarding T_H_-cell lineages at mRNA and cytokine protein level in relation to T_H_1 genotypes in a well documented birth cohort, including the follow-up for atopic diseases until the age of 3 years. It reveals that polymorphisms in crucial transcription factors of T_H_1 cells lead to alterations in the T_H_2 pathway already in early life. The further follow-up of our cohort will help to elucidate which of these children will also develop atopic diseases and/or asthma and whether the *TBX21* and *HLX* genotypes have an impact on their later immune development.

## Supporting Information

Table S1 **Distribution of** ***TLR2*** **polymorphisms within** ***HLX1*** **genotypes.** Results are presented as % and below absolute numbers shown in brackets.
(DOC)
