# Supplementary material for: TBX21 and HLX1 Polymorphisms Influence Cytokine Secretion at Birth
Source: PLoS One. 2012 Jan 30;7(1):e31069. doi: 10.1371/journal.pone.0031069 (PMC3268767; doi:10.1371/journal.pone.0031069)
Supplement: Table S1 — Distribution of TLR2 polymorphisms within HLX1 genotypes. Results are presented as % and below absolute numbers shown in brackets. (DOC) [file pone.0031069.s001.doc]

**Table S1. Distribution of *TLR2* polymorphisms within *HLX1* genotypes.**

| **Gene/rs number** | **Genotype** | ***TLR2* rs1898830** | | | ***TLR2* rs4696480** | | |
| --- | --- | --- | --- | --- | --- | --- | --- |
|  |  | **WT** | **HT** | **SNP** | **WT** | **HT** | **SNP** |
| ***HLX1* rs2738751** | **WT** | 40.26 (31/77) | 46.75 (36/77) | 12.99 (10/77) | 23.53 (32/136) | 47.79 (65/136) | 28.68 (39/136) |
|  | **HT** | 51.85 (14/27) | 40.74 (11/27) | 7.41 (2/27) | 17.39 (8/46) | 52.17 (24/46) | 30.43 (14/46) |
|  | **SNP** | 33.33 (1/3) | 33.33 (1/3) | 33.33 (1/3) | 50 (2/4) | 25 (1/4) | 25 (1/4) |
| ***HLX1* rs12141189** | **WT** | 45.90 (28/61) | 44.36 (27/61) | 9.84 (6/61) | 22.64 (24/106) | 48.11 (51/106) | 29.25 (31/106) |
|  | **HT** | 39.53 (17/43) | 48.84 (21/43) | 11.63 (5/43) | 20 (14/70) | 51.43 (36/70) | 28.57 (20/70) |
|  | **SNP** | 25 (1/4) | 25 (1/4) | 50 (2/4) | 33.33 (4/12) | 41.67 (5/12) | 25 (3/12) |
